# Spatio-temporal regulation of Wnt and retinoic acid signaling by *tbx16/spadetail *during zebrafish mesoderm differentiation

**DOI:** 10.1186/1471-2164-11-492

**Published:** 2010-09-09

**Authors:** Rachel Lockridge Mueller, Cheng Huang, Robert K Ho

**Affiliations:** 1 Department of Organismal Biology and Anatomy, The University of Chicago, Chicago IL 60637, USA; 2 Department of Biology, Colorado State University, Fort Collins CO 80523, USA

## Abstract

**Background:**

A complex network of signaling pathways and transcription factors regulates vertebrate mesoderm development. Zebrafish mutants provide a powerful tool for examining the roles of individual genes in such a network. *spadetail (spt) *is a mutant with a lesion in *tbx16*, a T-box transcription factor involved in mesoderm development; the mutant phenotype includes disrupted primitive red blood cell formation as well as disrupted somitogenesis. Despite much recent progress, the downstream targets of *tbx16 *remain incompletely understood. The current study was carried out to test whether any of the five major signaling pathways are regulated by *tbx16 *during two specific stages of mesoderm development: primitive red blood cell formation in the intermediate mesoderm and somite formation in the tail paraxial mesoderm. This test was performed using Gene Set Enrichment Analysis, which identifies coordinated changes in expression among *a priori *sets of genes associated with biological features or processes.

**Results:**

Our Gene Set Enrichment Analysis results identify Wnt and retinoic acid signaling as likely downstream targets of *tbx16 *in the developing zebrafish intermediate mesoderm, the site of primitive red blood cell formation. In addition, such results identify retinoic acid signaling as a downstream target of *tbx16 *in the developing zebrafish posterior somites. Finally, using candidate gene identification and *in situ *hybridization, we provide expression domain information for 25 additional genes downstream of *tbx16 *that are outside of both pathways; 23 were previously unknown downstream targets of *tbx16*, and seven had previously uncharacterized expression in zebrafish.

**Conclusions:**

Our results suggest that (1) *tbx16 *regulates Wnt signaling in the developing zebrafish intermediate mesoderm, the site of primitive red blood cell formation, and (2) *tbx16 *regulates retinoic acid signaling at two distinct embryonic locations and developmental stages, which may imply ongoing spatio-temporal regulation throughout mesoderm development.

## Background

Vertebrate mesoderm development is directed by a complex network of signaling pathways and transcription factors [1-6]. Most of the major signaling pathways -- TGF-β, FGF, Wnt, Delta-Notch, and retinoic acid -- have been identified, and many of their interactions have been elucidated. For example, Nodal, BMP, Wnt, and FGF pathways communicate in complex ways to specify both cell fate and cell movement during gastrulation [4]; Wnt, FGF, and Delta-Notch pathways interact with associated transcription factors to direct segmentation [7]; and BMP, Notch, and Wnt pathways interact with associated transcription factors to regulate blood and vessel formation [8]. However, despite much progress, the diverse ways in which these pathways interact to regulate cell fate and morphogenesis remain an area of intense research [1], and many more such interactions likely remain uncharacterized.

Analysis of mutants displaying specific mesodermal defects is a powerful tool with which to study the molecular basis of mesoderm specification and morphogenesis -- the more limited the scope of the mutant phenotype, the more focused the analysis of underlying molecular mechanisms that can be performed. *spadetail (spt) *is a zebrafish mutant with a lesion in *tbx16*, a T-box gene involved in mesoderm development [9,10]. T-box genes are a family of transcription factors, distinguished by a DNA binding domain (the "T-box"), that regulate numerous developmental processes; the gene family likely arose in the common ancestor of metazoans, and vertebrates possess approximately 20 members [11]. *spt *mutants lack trunk paraxial mesoderm because the appropriate mesodermal precursor cells mismigrate during gastrulation, localizing in the tailbud instead of converging dorsally to populate the trunk [12]. *spt *mutants have severely compromised primitive and definitive red blood cell formation [13,14] and irregular vasculature [15]; in some cases, they lack pectoral fins, an anus, and a pronephric kidney [14]. While trunk somites are absent, somewhat irregular tail somites do form in *spt *mutants [14], concomitant with intact segmentation clock gene expression machinery [16]. Other mesoderm-derived tissues develop largely normally. Thus, the zebrafish *spt *mutant is an excellent system in which to examine the molecular mechanisms underlying specific mesoderm-derived structures in an otherwise largely unaffected embryo.

*tbx16 *is embedded in the network of signaling pathways and transcription factors involved in mesoderm development, although not initial mesoderm induction [17,18]. *tbx16 *expression is maintained by FGF signaling from the mid-gastrula stage onward, and it also becomes dependent on the T-box transcription factor *no tail *(*ntl*) during somitogenesis. *tbx16*, in combination with *ntl *and its paralog *bra*, in turn regulates expression of the T-box transcription factor *tbx6 *in the trunk [9,19,20]. Additive, antagonistic, and combinatorial interactions among *ntl*, *tbx6*, and *tbx16 *direct cell fate specification in the developing mesoderm [21], and the specific downstream targets/pathways mediating such specification are incompletely understood and remain the focus of intense study [19].

The role of *tbx16 *in specifying embryonic (primitive) red blood cell fate has been examined in some detail [13,22,23]. *spt *mutants lack red blood cell-specific gene expression (e.g. *αe1globin*, *βe1globin*, *gata1 *and *jak2a*), indicating that red blood cell differentiation is not initiated [22]. In addition, expression of early hematopoietic genes associated with blood progenitor formation is absent, delayed, and/or downregulated in *spt *[22], indicating that *tbx16 *acts prior to hematopoietic stem cell specification. Significantly, transplant experiments indicate that a novel signaling interaction between paraxial and intermediate mesoderm necessary for specifying primitive red blood cell fate is missing in *spt *mutants [22]. Thus, *tbx16 *appears to play an additional, as-yet-uncharacterized role in the network of transcription factors and signaling pathways necessary to specify mesoderm fate. The current study was carried out to test whether any of the five major signaling pathways are regulated by *tbx16 *during two specific stages of mesoderm development: primitive red blood cell formation in the intermediate mesoderm and somite formation in the tail.

As in other model organisms, microarray data have been used in zebrafish to generate transcriptome profiles during embryonic development [24] as well as to explore the downstream effects of mutant alleles [23,25,26]. Gene Set Enrichment Analysis (GSEA), applied to microarray data, identifies coordinated changes in expression among *a priori *sets of genes associated with biological features or processes [27,28]. GSEA has been used to investigate zebrafish models of human cancers [29-31] and more general questions in developmental genomics [32]; however, this technique has not been widely used to examine the effects of specific mutations during zebrafish development. Here, we use GSEA to detect the *spt*-mediated disruption of groups of genes comprising different signaling pathways. We used both dissection and fluorescence activated cell sorting (FACS) to isolate different mesodermal tissues within the developing zebrafish embryo for genomic analysis. We present (1) GSEA analyses of two developing mesoderm tissues that test for up- or downregulation of the five major signaling pathways. Such analyses identify Wnt and retinoic acid signaling as likely downstream targets of *tbx16 *in one or both developing tissues, respectively; (2) microarray-based analyses of individual downstream target genes of *tbx16 *in both tissues; and (3) *in situ *hybridization-based exploration of our microarray results, which yielded expression domain data for 26 genes downstream of *tbx16*. Twenty-five such genes are outside of either disrupted signaling pathway, 23 were previously unknown downstream targets of *tbx16*, and seven had previously uncharacterized expression in zebrafish. More generally, our results support the utility of GSEA in zebrafish developmental genomics [32].

## Methods

### Fish Strains

Fish were cared for and handled with standard techniques [33]. Zebrafish harboring the recessive *b104 *mutant allele of the *spt/tbx16 *gene were crossed with a transgenic line carrying the *pax2a *(previously *pax2.1*) promoter fused to a GFP reporter gene [34]. Resulting offspring were used for all microarray and *in situ *hybridization analyses; *spt*^+/+ ^embryos ("wild-type," hereafter) were used as controls for comparison with *spt *^b104/b104 ^embryos ("*spt" *hereafter) in the microarray analyses, and both *spt^+/+ ^and spt*^b104/+ ^were used as "wild-type" controls for *in situ *hybridization analyses. *pax2a*-GFP positive fish were used for 4/5-somite microarray analyses; 21-somite microarray analyses and all *in situ *hybridization analyses used fish unsorted for *pax2a*-GFP. All experiments and animal husbandry were carried out in accordance with standards set by The University of Chicago's Animal Care and Use Committee (Protocol # 71112 to R. K. Ho).

### Tissue Dissection, Fluorescence-Activated Cell Sorting, and RNA extraction

RNA was extracted for microarray analysis at two developmental stages: 4/5 somites (approximately 11.5 hours post-fertilization, or hpf) and 21 somites (approximately 19.5 hpf). The 4/5-somite stage analysis targeted the intermediate mesoderm, the site of *tbx16-*dependent erythropoiesis. The 21-somite stage analysis targeted the paraxial mesoderm, where *tbx16 *is required for normal somitogenesis, but the tissue used for RNA extraction also included other tissue types (e.g. intermediate mesoderm, notochord, spinal cord). Each RNA extraction procedure was repeated three times from separate clutches of embryos. Embryos were staged following Kimmel et al. (1995) [35]. At the 4/5-somite stage, *pax2a*-GFP positive embryos were dissected in Hanks' solution; everything anterior to the first somite, as well as the majority of the yolk, was removed to eliminate fluorescence associated with structures other than the intermediate mesoderm. The remainder of the embryo was minced. Minced tissue from eight embryos was combined and dissociated in 1.2 mL of 0.15% Trypsin (Sigma) and 2.4 U/mL Dispase (Gibco) for one hour at room temperature with constant stirring. Cells were passed through a 40 μm cell strainer, pelleted, and resuspended in Hanks'. Cells were sorted using a DakoCytomation MoFlo-HTS cell sorter. Approximately 50 - 100 GFP-positive cells from each sample were collected into 100 μL Buffer XB (Arcturus). To the extent possible, the same numbers of wild-type and *spt *cells were used as starting material in each of the three replicates. Because fluorescent cell numbers were so low, a portion of the sorted cell population was not re-run through the instrument to quantify sort purity, as is routinely done; however, the instrument was calibrated prior to each run using beads to ensure that it was sorting at >99% purity. Dead and dying cells were excluded using the FS vs. SS morphology FACS profile. RNA was extracted using the PicoPure Isolation Kit (Arcturus) according to the manufacturer's protocol. Two rounds of RNA amplification were carried out using the RiboAmp HS RNA Amplification Kit (Arcturus) according to the manufacturer's instructions. At the 21-somite stage, embryos were dissected in Daniaeu's media; tissue including the somites and pre-somitic mesoderm posterior to the yolk-sac extension was collected from 150 wild-type and *spt *embryos for each replicate. The posterior-most tip of the tail in the wild-type embryos and the abnormal ball of cells at the tip of the tail in the *spt *embryos were removed. RNA was extracted using the PicoPure Isolation Kit (Arcturus) according to the manufacturer's protocol. RNA integrity for both amplified and unamplified samples was assessed using an Agilent 2100 Bioanalyzer (Agilent Technologies, Palo Alto, CA).

### Microarray Hybridization and Gene Set Enrichment Analyses

Biotinylated cRNA was prepared from ~1-2 μg total RNA (21-somite stage) or ~6 μg amplified RNA (4/5-somite stage) and hybridized to Affymetrix GeneChip^® ^Zebrafish Genome Arrays by The University of Chicago functional genomics core facility according to the manufacturer's protocol. Image acquisition and initial array quantification were performed using the Affymetrix Microarray Suite Version 5.0. Gene sets for signaling pathways and specific tissues were compiled from the following sources: KEGG (Kyoto Encyclopedia of Genes and Genomes) Pathway Database Section 3.2 (Environmental Information Processing > Signal Transduction) http://www.genome.jp/kegg/pathway.html; the Wnt Homepage (R. Nusse, http://www.stanford.edu/~rnusse/wntwindow.html; the gene ontology information in the Affymetrix zebrafish array annotation; the ZFIN anatomical ontology browser http://zfin.org; OMIM http://www.ncbi.nlm.nih.gov/omim/; and the literature. Only genes represented on the array were included in gene sets for GSEA. For signaling molecules that activate multiple pathways (e.g. non-canonical vs. canonical pathways activated by *wnt *ligands), the multiple pathways were combined into single gene sets for GSEA; gene sets constructed for separate pathways yielded gene sets too short for analysis. Gene sets ranged from 15 genes/splice variants (retinoic acid signaling list) to 194 genes/splice variants (somite list) and are included as Additional file 1. Tissues from the two developmental stages were analyzed separately. Affymetrix .CEL files for the 21-somite arrays (wild-type and *spt*) were combined for enrichment analysis using the ExpressionFileCreator module of the GenePattern software package (quantile normalization = yes, background correct = yes), as were the 4/5-somite arrays (wild-type and *spt*). GSEAs for the five signaling pathways and the positive control tissue(s) were run using GSEA software http://www.broad.mit.edu/gsea/ at the probe level (Collapse dataset to gene symbols = false) with 1,000 gene set permutations. Genes in the expression datasets were ranked using the Signal2Noise metric (21-somite analysis) and log_2 _Ratio of Classes metric (4/5-somite analysis) following GSEA recommendations for our sample sizes. Probes for several genes were included in ≥ two gene sets at both developmental stages: *dvl2 *and *plcg1 *for the 4/5-somite analysis, and *dlc*, *dvl2*, *fgf8*, *fst*, *jag2, lfng*, *map3k4*, *plcg1*, *sfrp5*, *smad1*, *wnt5b*, and *wnt11 *for the 21-somite analysis. Because the inclusion of genes in multiple gene sets can affect statistical significance levels, analyses were repeated both including and excluding these twelve genes. Significance was defined at the False Discovery Rate (FDR) q-value = 0.10 level, following suggestions by the GSEA creators that smaller datasets analyzed with gene set permutation adopt a more conservative FDR q-value cutoff than the default 0.25.

Because the zebrafish genome annotation is incomplete, GSEA of this array must contend with two potential problems: (1) multiple probe sets specifying the same gene (EST cluster), and (2) unannotated probes. We addressed these potential problems in several ways. First, to address probe redundancy, we took the following measures: (1) elimination from the gene set lists of all redundant probes with known cross-hybridization signal (denoted by _x_in Affymetrix notation), and (2) elimination from the gene set lists of the non-expressed probe when both sense and antisense probes exist for the same EST subcluster. Redundant probes specifying different EST subclusters and/or alternative transcripts were all included in the analysis. Second, to address incomplete probe annotation, we included in our analysis two gene sets that describe tissues known to be reduced or abnormal in the *spt *mutant. These gene sets serve as positive controls, as they test whether the signal contained in the zebrafish array is sufficient to be detected by GSEA despite the noise of unannotated probes, as well as any other sources of experimental noise inherent in the microarray data. These positive control gene sets contain genes associated with red blood cells and somites. Red blood cell markers are expected to be present in the wild type and downregulated in *spt *in both 4/5- and 21-somite-stage tissues. Somite markers (specifically somites 20-25) are expected to be present in the wild type and perturbed in *spt *in the 21-somite-stage tissue. Microarray results are deposited in the Gene Expression Omnibus (Series # GSE19955).

### Candidate Gene Identification and *In Situ *Hybridization

**Figure 1:**
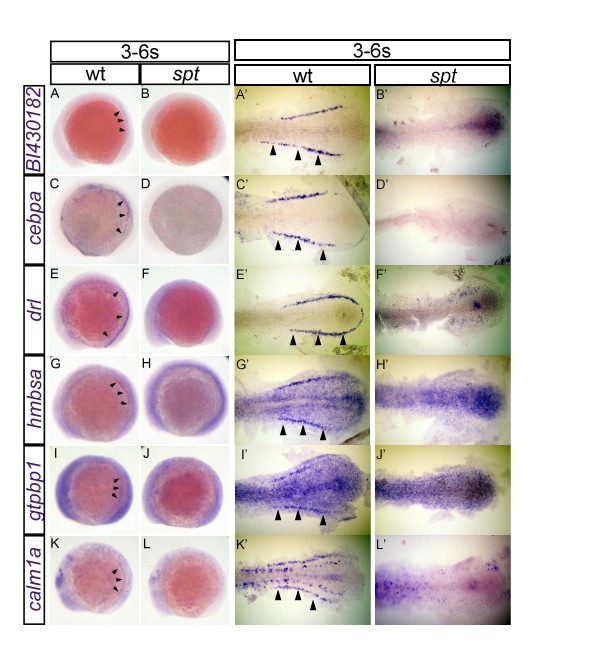
**Expression of validated candidate genes from the 4/5-somite microarray**. Changes in the expression of *BI430182 *(A, B, A', B'), *cebpa *(C, D, C', D'), *drl *(E, F, E', F'), *hmbsa *(G, H, G', H'), *gtpbp1 *(I, J, I', J') and *calm1a *(K, L, K', L') were visualized using *in situ *hybridization in wild-type (A, A', C, C', E, E', G, G', I, I', K and K') and *spt *embryos (B, B', D, D', F, F', H, H', J, J', L and L'). All embryos are oriented with posterior to the right. Whole-mount embryos (A-L) are shown in lateral view while de-yolked and flat-mounted embryos (A'-L') are shown in dorsal view. Flat-mounted embryos (A'-L') show only the posterior half of the embryos for high-magnification view. All embryos were fixed at the 3 to 6-somite stage. Arrowheads point to specific intermediate mesoderm expression domains.

Individual genes with different expression patterns in wild-type vs. *spt *embryos were identified using the dCHIP [36] and GeneSpring (Agilent Technologies, Santa Clara, CA) software packages to achieve two aims: (1) select genes whose array-based expression differences would be corroborated using different methods, and (2) identify specific downstream targets of *tbx16 *involved in mesoderm development, either linked to or outside of any identified signaling pathways. Groups of arrays from the two developmental stages were analyzed independently. Arrays were normalized using invariant set normalization. Analyses were run on both filtered and unfiltered data. For filtered analyses, probe sets with expression levels below 100 in ≥ 50% of samples, or called as "absent" in ≥ 80% of samples, were excluded from further analyses. For unfiltered analyses, all probe sets were included. One wild-type 4/5-somite stage array was excluded from both candidate gene and GSEA analyses because it did not cluster with the other wild-type replicates (GeneSpring > Find Similar Samples analysis); further investigation of the expression of known zebrafish red blood cell markers (e.g. *gata1*) in this anomalous array showed expression levels up to two orders of magnitude lower than in the other two wild-type arrays. Genes that displayed a ≥ 1.25-fold (21-somite microarray) or ≥ 1.5-fold (4/5-somite microarray) change in expression between wild-type and *spt *embryos were identified at each developmental stage (two-tailed p < 0.05, median false discovery rate determined with 720 permutations). Distributions of p-values were examined to aid in interpreting false discovery rates. Forty-eight genes identified as differentially expressed between wild-type and *spt *tissues were screened by whole mount *in situ *hybridization at either the 3-6-somite stage (those identified by the 4/5-somite microarray) or the 21-somite stage (those identified by the 21-somite microarray) (Table 1). The following antisense riboprobes for *in situ *hybridization were synthesized as described: *drl *[37], *jak2a *[38], *zeb2a *(previously *sip1a*) [39], *hoxa11a *[40], *aldh1a2 *(previously *raldh2*) [41], and *hsp90a.1 *[42]. The following antisense riboprobe was synthesized using a clone purchased from the Zebrafish International Resource Center http://zebrafish.org/zirc/home/guide.php: *unc45b *(clone ID cb393). To make the rest of the riboprobes, clones were purchased from Thermo Scientific Open Biosystems http://www.openbiosystems.com. The Open Biosystem clone IDs for such clones are: 4967423 (*BI430182*), 7289896 (*zgc:171560*), 7418866 (*zgc:112524*), 7055776 (*zgc:92345*), 7400556 (*zgc:110288*), 6793849 (*cebpa*), 6796795 (*hmbsa*), 3817707 (*gtpbp1*), 6790866 (*calm1a*), 7270071 (*zgc:136864*), 7432799 (*zgc:153587*), 3818293 (*dnajc21*), 8122978 (*zgc:153390*), 5913990 (*ptgesl*), 7417087 (*zfyve21*), 3818920 (*ddx3*), 6797250 (*zgc:64161*), 5915334 (*rnaseh2b*), 5410935 (*nae1*), 7000848 (*zgc:77744*), 3820017 (*zgc:56033*), 6800295 (*zgc:66110*), 6791918 (*zgc:63962*), 6805707 (*atp6v1e1*), 5600729 (*pdap1*), 6961180 (*pinx1*), 7001512 (*exosc4*), 5411920 (*zgc:56136*), 4787610 (*znf593*), 7923686 (*LOC798291*), 4789934 (*zgc:73329*), 7157357 (*tcf25*), 8148733 (*clstn1*), 7223265 (*si:dkey-177p2.6*), 6792738 (*aldoc*), 7001080 (*slc1a3a*), 6893660 (*LOC568423*), 6894907 (*s1pr1*), 7036656 (*rcan3*), 7052011 (*bmp3*), and 6794698 (*fgfrl1a*). In the event that such clones contained no RNA polymerase sites flanking the inserts, inserts were sub-cloned into pBluescript using appropriate restriction enzymes before riboprobes were synthesized. *In situ *hybridization was performed as previously described [43] using NBT/BCIP as the enzyme substrates, except that 0.2× SSC was replaced with 0.05× SSC. In the cases of certain probes such as *gtpbp1 *and *calm1a*, expression level within the intermediate mesoderm is lower than that outside of the intermediate mesoderm. Hence, NBT/BCIP developing time was extended to allow visualization of the specific intermediate mesoderm expression, resulting in over-staining of expression domains outside of the intermediate mesoderm. Following *in situ *hybridization, some of the embryos were de-yolked and flat-mounted before photographs were taken (Figure 1, A'-L'). Although *in situ *hybridization is only a semi-quantitative measurement of differential expression, one goal of our study was to ascertain the usefulness of this method for identification of developmentally significant expression domain information; thus, this measurement was used in preference to more quantitative measurements for array corroboration (e.g. qPCR), which would not have provided region-specific differential expression information. Similar studies in zebrafish developmental genomics have shown the combination of microarray analysis and *in situ *hybridization to be an effective strategy for identifying differentially expressed genes between wild-type and mutant embryos [23,26].

**Table 1:** Summary of candidate genes identified from microarray analyses analyzed with *in situ *hybridization.

Affy Probe ID	Gene **Name**^**a**^	Gene Symbol	Corroborate **Microarray**^**b**^	Expression **Domain**^**c**^	Expression in ***spt *Embryos **^**d**^	Expression Studies Previously Reported	Identified by Array	Fold-Change in Array
Dr.16366.1.S1_at	*BI430182*^e^	*BI430182*^f^	Yes	IM	downregulated	No	4/5s	-5.54

Dr.14123.1.A1_at	*zgc:171560*	*zgc:171560*^f^	Yes	ubiquitous	downregulated	No	4/5s	-4.51

Dr.16573.3.S1_x_at	*zgc:112524*	*zgc:112524*^f^	Yes	ubiquitous	downregulated	No	4/5s	-3.49

Dr.12443.1.A1_at	*zgc:92345*	*zgc:92345*^f^	Yes	ubiquitous	downregulated	No	4/5s	-2.73

Dr.1085.2.A1_at	*zgc:110288*	*zgc:110288*^f^	Yes	ubiquitous	downregulated	No	4/5s	-2.45

Dr.12055.1.S1_at	*CCAAT/enhancer binding protein (C/EBP), alpha*	*cebpa*^f^	Yes	IM, ALPM	downregulated	Yes	4/5s	-16.67

Dr.8064.1.S1_at	*draculin*	*drl*	Yes	IM	downregulated	Yes	4/5 s and 21s	-15.64 and -3.65

Dr.3338.1.S1_at	*hydroxymethylb ilane synthase a*	*hmbsa*^f^	Yes	IM, midline structures	downregulated	Yes	4/5s	-4.56

Dr.515.1.A1_at	*GTP binding protein 1*	*gtpbp1*^f^	Yes	IM, midline structures	downregulated	Yes	4/5s	-2.68

Dr.7908.1.S2_at	*calmodulin 1a*	*calm1a*^f, g^	Yes	IM, neuroectoderm	downregulated	Yes	4/5s	-2.11

Dr.4151.1.S1_at	*Janus kinase 2a*	*jak2a*	Yes	ubiquitous	downregulated	Yes	4/5s	-4.64

Dr.21814.1.S1_at	*zgc:136864*	*zgc:136864*	No - Category A	ubiquitous	same	No	4/5s	-5.73

Dr.1118.1.A1_at	*zgc:153587*	*zgc:153587*	No - Category A	ubiquitous	same	No	4/5s	-4.44

Dr.883.1.A1_at	*DnaJ (Hsp40) homolog, subfamily C, member 21*	*dnajc21*	No - Category A	ubiquitous	same	No	4/5s	-2.52

Dr.14906.1.A1_at	*zgc:153390*	*zgc:153390*	No - Category A	ubiquitous	same	No	4/5s	-2.21

Dr.16120.1.S1_at	*prostaglandin E synthase 2-like*	*ptgesl*	No - Category A	ubiquitous	same	Yes	4/5s	-4.98

Dr.3023.1.S1_at	*zinc finger, FYVE domain containing 21*	*zfyve21*	No - Category A	ubiquitous	same	Yes	4/5s	-4.47

Dr.8412.1.A1_at	*DEAD (Asp-Glu-Ala-Asp) box polypeptide 3*	*ddx3*	No - Category A	ubiquitous	same	Yes	4/5s	-3.7

Dr.4269.1.A1_at	*zgc:64161*	*zgc:64161*	No - Category A	ubiquitous	same	Yes	4/5s	-3.56

Dr.4468.1.S1_at	*ribonuclease H2, subunit B*	*rnaseh2b*	No - Category A	ubiquitous	same	Yes	4/5s	-2.97

Dr.7212.1.S1_at	*nedd8 activating enzyme E1 subunit 1*	*nae1*	No - Category A	ubiquitous	same	Yes	4/5s	-2.84

Dr.12643.1.A1_at	*zgc:77744*	*zgc:77744*	No - Category A	ubiquitous	same	Yes	4/5s	-2.71

Dr.15269.1.S1_at	*zgc:56033*	*zgc:56033*	No - Category A	ubiquitous	same	Yes	4/5s	-2.68

Dr.14026.1.A1_at	*zgc:66110*	*zgc:66110*	No - Category A	ubiquitous	same	Yes	4/5s	-2.68

Dr.7237.1.S1_at	*zgc:63962*	*zgc:63962*	No - Category A	ubiquitous	same	Yes	4/5s	-2.51

Dr.7966.1.S1_at	*ATPase, H+ transporting, lysosomal, V1 subunit E isoform 1*	*atp6v1e1 *^f^	No - Category B	polster, neuroectoderm	downregulated	No	4/5s	-2.54

Dr.1791.1.S1_at	*pdgfa associated protein 1*	*pdap1 *^f^	No - Category B	polster	downregulated	Yes	4/5s	-4.25

Dr.14426.1.S1_at	*pin2/trf1-interacting protein 1*	*pinx1 *^f^	No - Category B	polster, neuroectoderm, PM	downregulated	Yes	4/5s	-3.04

Dr.9715.3.S1_a_at	*exosome component 4*	*exosc4 *^f^	No - Category B	PM	downregulated	Yes	4/5s	-3

Dr.7799.1.A1_at	*zgc:56136*	*zgc:56136*^f^	No - Category B	neuroectoderm	downregulated	Yes	4/5s	-2.73

Dr.18052.1.S1_at	*zinc finger protein 593*	*znf593*^f^	No - Category B	neuroectoderm, PM	downregulated	Yes	4/5s	-2.46

Dr.893.2.S1_at	*LOC798291*	*LOC798291*	No - Category C	PM	same	No	4/5s	-2.32

Dr.9423.1.S1_at	*zgc:73329*	*zgc:73329*	No - Category C	PM	same	Yes	4/5s	-3.25

Dr.1663.1.A1_at	*transcription factor 25 (basic helix-loop-helix)*	*tcf25*	No - Category C	polster	same	Yes	4/5s	-2.76

Dr.8822.1.A1_at	*zinc finger E-box binding homeobox 2a*	*zeb2a*	No - Category C	neuroectoderm	same	Yes	4/5s	-3.6

Dr.25212.1.A1_at	*calsyntenin 1*	*clstn1*	No - Category C	Kupffer's vesicle, tail bud	same	Yes	4/5s	-3.28

Dr.12545.1.S1_at	*si:dkey-177p2.6*	*si:dkey-177p2.6*	No - Category C	neuroectoderm, PM	same	Yes	4/5s	-2.87

Dr.19223.1.S2_at	*aldolase c, fructose-bisphosphate*	*aldoc*	No - Category C	neuroectoderm	same	Yes	4/5s	-5.23

Dr.25679.1.S1_at	*solute carrier family 1 (glial high affinity glutamate transporter), member 3a*	*slc1a3a*	No - Category C	neuroectoderm	same	Yes	4/5s	-3.05

Dr.1002.1.S1_at	*LOC568423*	*LOC568423 *^f^	No - Category D	ubiquitous	upregulated	No	4/5s	-2.34

Dr.25683.5.A1_at	*sphingosine-1-phosphate receptor 1*	*s1pr1*^f^	No - Category D	neuroectoderm, tailbud	upregulated	Yes	4/5s	-2.76

Dr.8183.1.S1_at	*homeo box A11a*	*hoxa11a*^f^	No - Category D	tailbud	upregulated	Yes	4/5s	-2.35

Dr.5206.1.S1_at	*aldehyde dehydrogenase 1 family, member A2*	*aldh1a2*	Yes	PM, eyes	downregulated	Yes	21s	-6.16

Dr.1817.1.A1_at	*regulator of calcineurin family member 3*	*rcan3*^f^	Yes	PM	downregulated	Yes	21s	-2.72

Dr.23348.1.A1_at	*bone morphogenetic protein 3*	*bmp3*^f, g^	Yes	PM, neuroectoderm	downregulated	Yes	21s	-1.68

Dr.610.1.S1_at	*heat shock protein 90-alpha 1*	*hsp90a.1 *^f, g^	Yes	PM	downregulated	Yes	21s	-2.26

Dr.345.1.S1_at	*unc-45 homolog B (C. elegans)*	*unc45b*^f, g^	Yes	PM	downregulated	Yes	21s	-2.39

Dr.26455.1.S1_at	*fibroblast growth factor receptor-like 1a*	*fgfrl1a*^f, g^	Yes	PM, eyes	downregulated	Yes	21s	-1.73

^a ^Gene names are consistent with those in ZFIN (The Zebrafish Model Organism Database, http://zfin.org/) whenever possible.

^b ^"Yes" is defined as intermediate mesoderm expression (for genes from the 4/5s microarray) or paraxial mesoderm expression (for genes from the 21s microarray) that is downregulated in *spt*. Category A is defined as ubiquitous expression that is not changed in *spt*. Category B is defined as expression that is downregulated in *spt *but that is absent from the intermediate mesoderm. Category C is defined as expression that is unchanged in *spt *and absent from the intermediate mesoderm. Category D is defined as expression upregulated in *spt*.

^c ^Abbreviations are ALPM (anterior lateral plate mesoderm), IM (intermediate mesoderm) and PM (paraxial mesoderm).

^d ^Downregulated is defined as at least part of the expression domain being reduced compared to the wild type based on visual detection. Upregulated is defined as at least part of the expression domain being enhanced, and no part of the expression domain being reduced, compared to the wild type, based on visual detection.

^e ^EST ID is used here because this Affy probe is annotated by EST only and has not been assigned to an annotated gene. All of the other candidates explored by *in situ *hybridization have been linked to annotated genes.

^f ^These genes, to our knowledge, have not previously been revealed to act downstream of *tbx16*.

^g ^These genes were identified as differentially expressed by the unfiltered analyses only; all other candidates explored by *in situ *hybridization were identified by the filtered analyses.

## Results

### Gene Set Enrichment Analysis

GSEA [28] was performed to determine whether specific signaling pathways specified *a priori *were altered in *spt *at different developmental stages. The following five major signaling pathways were examined: (1) Wnt, (2) Delta-Notch, (3) TGF-β, (4) retinoic acid, and (5) FGF.

#### 4/5-Somite Intermediate Mesoderm Tissue Analysis

As described above, intermediate mesoderm cells were isolated by FACS sorting of *pax2a*-GFP positive cells in both otherwise wild-type and *spt *mutant embryos. The wild-type tissues contained slightly higher relative numbers of GFP-positive cells (1.5% - 5%) than did the *spt *tissues (0.38% - 3.2%); variation among runs likely reflects variable fluorescence levels among individual *pax2a*-GFP fish. The red blood cell gene set (Additional file 1), which serves as our positive control, is significantly downregulated in *spt *as expected based on the mutant phenotype (FDR q = 0.000, NES = - 2.468). This implies that (1) the zebrafish array annotation is sufficient to provide information at this level of analysis and (2) the array data contain a sufficiently high signal-to-noise ratio for GSEA. The Wnt signaling pathway gene list is significantly upregulated in *spt *relative to wild-type tissue (FDR q = 0.034, NES = 1.574) and the retinoic acid pathway is significantly downregulated in *spt *(FDR q = 0.067, NES = -1.344). The other three signaling pathways examined are not significantly up- or downregulated in *spt *4/5-somite intermediate mesoderm tissue at the FDR q = 0.1 level (TGF-β FDR q = 0.415, NES = 1.110; Delta-Notch FDR q = 0.455, NES = 1.171; FGF FDR q = 0.349, NES = 1.081). Reanalysis excluding the two genes present in multiple gene sets yields similar results (red blood cell FDR q = 0.0, NES = -2.470; Wnt FDR q= 0.029, NES = 1.558; retinoic acid FDR q = 0.069, NES = -1.333; TGF-β FDR q = 0.415, NES = 1.110; Delta-Notch FDR q = 0.455, NES = 1.171; FGF FDR q = 0.350, NES = 1.081). Fifteen of the 60 total genes (16 of 70 total probes) comprising the Wnt gene list contribute to the leading-edge subset, whose expression levels drive the signal of Wnt upregulation in *spt*; 14 of these genes (14 of 66 total probes) contribute to the leading-edge subset when genes present in multiple gene sets are excluded (Additional file 2). Two of the 15 total genes (four of 19 total probes) comprising the retinoic acid gene list contribute to the leading-edge subset; this result is identical both including and excluding genes present in multiple gene sets (Additional file 2).

#### 21-Somite Tail Tissue Analysis

In *spt *embryos, trunk somitic mesoderm is largely absent due to the mis-migration of mesodermal cells during gastrulation [12]. However, in the *spt *tail region (defined roughly as the area behind the hindyolk where somites 17-32 would normally reside in wild-type embryos), somitic mesoderm structures are present. Isolation of the entire middle tail region, as outlined in the methods section, allowed us to compare gene expression profiles from a variety of tissues in wild-type versus *spt *embryos. Both the red blood cell gene set and the somite gene set (Additional file 1), which serve as positive controls, are significantly downregulated in *spt *as expected based on the mutant phenotype (red blood cell FDR q = 0.000, NES = -2.660; somite FDR q = 0.000, NES = -2.731); thus, these array data also contain a sufficiently high signal-to-noise ratio for GSEA analysis, and annotation is sufficient. The retinoic acid signaling pathway is significantly downregulated in *spt *relative to wild-type tissue (FDR q = 0.049, NES = -1.410). No other signaling pathway gene lists are significantly up- or downregulated in *spt *for the 21-somite tail tissue (Wnt FDR q = 0.615, NES = 1.091; TGF-β FDR q = 0.609, NES = 1.000; Delta-Notch FDR q = 0.673, NES = 0.889; FGF FDR q = 0.793, NES = 1.173). Reanalysis excluding the 12 genes present in multiple gene sets yields similar results (red blood cell FDR q = 0.000, NES = -2.627; somite FDR q = 0.000, NES = 2.735; retinoic acid FDR q = 0.057, NES = -1.382; Wnt FDR q = 1.0, NES = 1.137; TGF-β FDR q = 0.641, NES = 0.902; Delta-Notch FDR q = 0.712, NES = 0.955; FGF FDR q = 0.832, NES = 1.020).

In summary, GSEA results from both 4/5- and 21-somite analyses share significant downregulation of the retinoic acid pathway as well as red blood cell genes, which serve as a positive control for the analyses; however, the two results differ in that the Wnt pathway is significantly upregulated in the 4/5-somite, but not in the 21-somite, analysis.

### Candidate Gene Identification and *In Situ *Hybridization

In addition to analyses of signaling pathway sensitivity to the *spt *mutation, candidate gene identification analyses were performed to search for individual downstream target genes of *tbx16 *involved in mesoderm development, either linked to or outside of Wnt and retinoic acid signaling, as well as to explore the reliability of the microarray results. Candidate gene analysis comparing wild-type and *spt *tissue at the 4/5-somite stage yielded 182 genes (median false discovery rate = 60.4%) and 112 genes (median false discovery rate = 59.8%) for unfiltered and filtered analyses, respectively. Candidate gene analysis comparing wild-type and *spt *tissue at the 21-somite stage yielded 239 genes (median false discovery rate = 3.8%) and 58 genes (median false discovery rate = 1.7%) for unfiltered and filtered analyses, respectively. A high FDR, such as that characterizing the 4/5-somite analysis, can signify either clean data with a distribution of p-values in which no more genes have p ≤ 0.05 than would be expected by chance (as is the case for the 4/5-somite analysis), or noisy data, which particularly impacts candidate selection based on p-values [44]. All gene lists are included in Additional file 3.

A subset of these candidates was screened by *in situ *hybridization to (1) perform semi-quantitative corroboration of the array results, and (2) further evaluate potential candidates for functional studies (presented elsewhere) based on expression domains. A total of 48 genes were screened, 41 of which were identified by the 4/5-somite array analyses, six of which were identified by the 21-somite array analyses, and one of which was identified by both; our emphasis on the 4/5-somite array analyses reflects both the significant GSEA results for Wnt and retinoic acid signaling as well as the extremely high false-discovery rate of the candidate gene analysis. Genes downregulated in *spt *were chosen because of the demonstrated roles of T-box genes as transcriptional activators [45,46]; fold-changes for differential expression between *spt *and wild-type ranged from -1.68 to -16.67 among the 48 genes. *In situ *hybridization results confirm a high false-positive rate for the 4/5-somite microarray and a low false-positive rate for the 21-somite microarray; "false-positive" is defined as significantly lower expression in *spt *compared to wild-type embryos in the intermediate mesoderm (for the 4/5-somite microarray) or the tail paraxial mesoderm (for the 21-somite microarray) detected by microarray analysis but not by *in situ *hybridization. We note that we did not include any compound in our FACS sorting to exclude 100% of dead and dying cells (e.g. propidium iodide), which may have contributed to the high false-positive rate of the 4/5-somite microarray; however, we did use the FS vs. SS morphology profile, which allowed the exclusion of many such unwanted cells.

#### *In Situ *Hybridization of 4/5-Somite Array Candidates

We performed *in situ *hybridization with embryos at the 3-6-somite stage for 42 genes chosen from the 4/5-somite microarray results (Table 1). Twenty-six percent (11/42) of the genes identified as downregulated in *spt *intermediate mesoderm by the 4/5-somite array analysis were also shown to have reduced expression in *spt *intermediate mesoderm by *in situ *hybridization (Table 1, lines 1-11; Figure 1); 33% (14/42) were shown to have equivalent expression between wild-type and *spt *intermediate mesoderm (Table 1, Category A); 14% (6/42) were shown to have lower expression in *spt *than in the wild type, but expression was only detected outside of the intermediate mesoderm (Table 1, Category B); 19% (8/42) were shown to have equivalent expression between *spt *and the wild type, but expression was only detected outside of the intermediate mesoderm (Table 1, Category C); and 7% (3/42) were shown to have higher expression in *spt *than in the wild type, contrary to the array results (Table 1, Category D). We note that RNA amplification can double the relative noise in microarray analyses by distorting expression ratios between control and experimental tissues [47]; thus, we cannot be sure that all downregulated genes identified by the array would have sufficiently different expression levels between wild-type and *spt *tissues to be detectable by *in situ *hybridization. Because of this, Category A and C genes do not clearly refute the array results, although they do not corroborate them, either. Category B and C genes, which do not show discernible expression in the intermediate mesoderm by *in situ *hybridization, may reflect imperfect cell sorting; however, Category B genes have reduced expression in *spt*, consistent with the array results. Category D genes are inconsistent with the array results. Taken together, these *in situ *results confirm a high false-positive rate for the array analysis. However, the false positive rate begins to decline as fold-change increases above 4-fold. This suggests that accuracy of the 4/5-somite intermediate mesoderm microarray analysis improves for larger fold-changes. Noise preferentially impacting smaller fold-changes, particularly if it is directionally unbiased, is less likely to mislead GSEA, which focuses on detecting gene sets that are over-represented at the extremes of the overall fold-change distribution in a dataset [28]. In total, three lines of evidence suggest the presence of detectable signal in the 4/5-somite dataset, despite such high levels of noise: (1) downregulation of our positive control gene set (red blood cell-specific genes) was highly significant; (2) false-positives declined as fold-changes increased above 4-fold, indicating that our 4/5-somite array analyses can more accurately detect larger fold-changes; and (3) independent lines of evidence from other experimental systems are also beginning to link Wnt signaling with blood formation [48-50]. These results confirm that GSEA can identify broader patterns of coordinated gene expression, despite low signal and/or significant noise in microarray data [27,28].

Despite the noise associated with candidate gene selection, our *in situ *hybridization results confirm specific intermediate mesoderm expression of *cebpa *(Figure 1c-d') [51] and *drl *(Figure 1e-f') [37]. We also report specific intermediate mesoderm expression of *BI430182 *(Figure 1a-b'), *hmbsa *(Figure 1g-h'), *gtpbp1 *(Figure 1i-j'), and *calm1a *(Figure 1k-l'), whose intermediate mesoderm expression at the 3-6-somite stage was not previously revealed. In all six cases, expression is reduced in *spt*, suggesting that they are downstream targets of *tbx16*. Five of these six genes were not previously understood to function downstream of *tbx16*, while a sixth (*drl*) has previously been implicated to act downstream of *tbx16 *in red blood cell specification in the zebrafish intermediate mesoderm [22]. In addition, we report expression domain data for 11 genes whose expression has not previously been reported, six of which are also novel downstream targets of *tbx16*: (1) *zgc*:*171560*, (2) *zgc:112524*, (3) *zgc:92345*, and (4) *zgc:110288 *have ubiquitous expression domains and are novel downstream targets of *tbx16 *with reduced expression in *spt*. (5) *atp6v1e1 *is expressed in the polster and neuroectoderm and is a novel downstream target of *tbx16 *with reduced expression in *spt*. (6) *LOC568423 *has ubiquitous expression and is a novel downstream target of *tbx16 *with increased expression in *spt*. (7) *zgc:136864*, (8) *zgc:153587*, (9) *dnajc21*, and (10) *zgc:153390 *have ubiquitous expression domains with no apparent expression perturbation in *spt *detectable by *in situ *hybridization. (11) *LOC798291 *is expressed in paraxial mesoderm with no apparent expression perturbation in *spt *detectable by *in situ *hybridization. Finally, we report seven additional genes whose wild-type expression was previously known, but that we reveal to be downstream targets of *tbx16*: (1) *pdap1 *is expressed in the polster and is a novel downstream target of *tbx16 *with reduced expression in *spt*. (2) *pinx1 *is expressed in the polster, neuroectoderm, and paraxial mesoderm and is a novel downstream target of *tbx16 *with reduced expression in *spt*. (3) *exosc4 *is expressed in the paraxial mesoderm and is a novel downstream target of *tbx16 *with reduced expression in *spt*. (4) *zgc:56136 *is expressed in the neuroectoderm and is a novel downstream target of *tbx16 *with reduced expression in *spt*. *(5) znf593 *is expressed in the neuroectoderm and paraxial mesoderm and is a novel downstream target of *tbx16 *with reduced expression in *spt*. (6) *s1pr1 *is expressed in the neuroectoderm and tail bud and is a novel downstream target of *tbx16 *with increased expression in *spt*. (7) *hoxa11a *is expressed in the tail bud and is a novel downstream target of *tbx16 *with increased expression in *spt*. We note that our current analysis cannot distinguish between differential expression in the *spt *mutant caused by different expression levels in the same number of cells or different numbers of cells expressing such genes at the same level. In summary, our *in situ *hybridization results reveal that candidate selection based solely on p-values for RNA amplified from a few (50-100) intermediate mesoderm cells at the 4/5-somite stage yields high false positive rates, although interesting candidate genes were still identified for follow-up using expression data. Overall trends toward increased accuracy with higher fold-changes suggest that more global analyses of coordinated gene expression (e.g. GSEA) are still likely to be able to extract meaningful biological signal from such data; the detection of our positive control (red blood cell) gene set by GSEA further corroborates this.

#### *In Situ *Hybridization of 21-Somite Candidates

**Figure 2:**
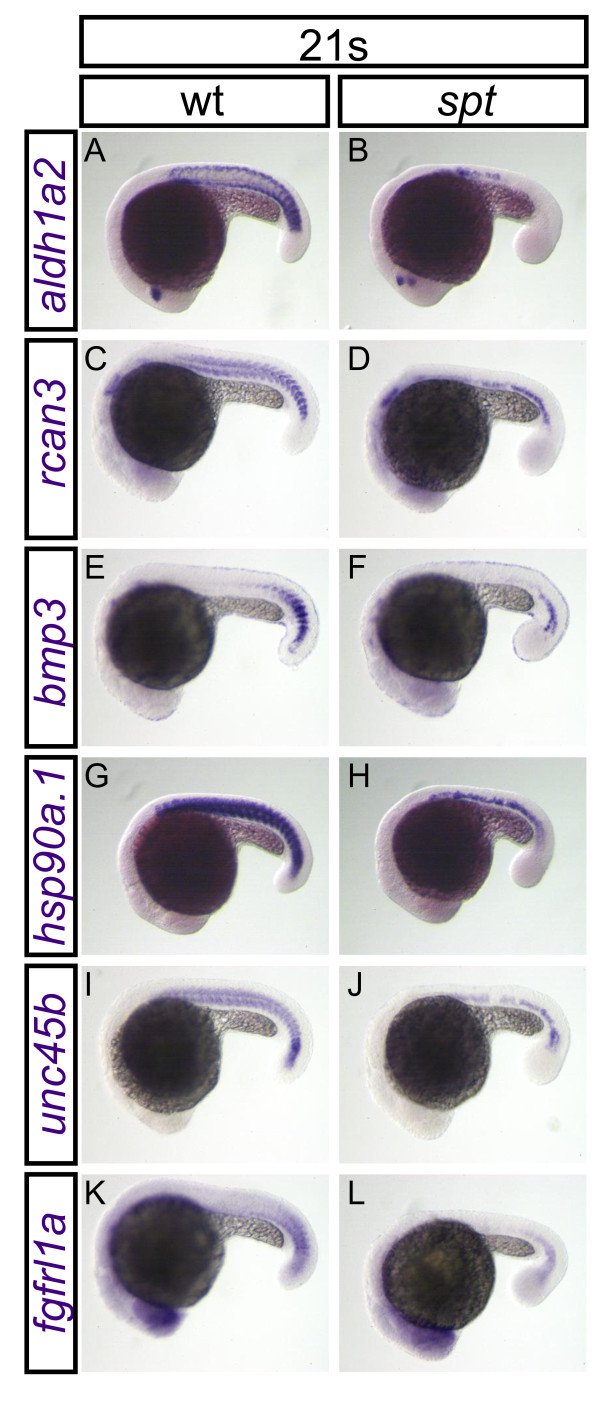
**Expression of validated candidate genes from the 21-somite microarray**. Changes in the expression of *aldh1a2 *(A, B), *rcan3 *(C, D), *bmp3 *(E, F), *hsp90a*.1 (G, H), *unc45b *(I, J) and *fgfrl1a *(K, L) were visualized using whole mount *in situ *hybridization in wild-type embryos (A, C, E, G, I and K) and *spt *embryos (B, D, F, H, J and L). All embryos are shown in lateral view with anterior to the left. All embryos were fixed at the 21-somite stage.

We performed *in situ *hybridization with embryos at the 21-somite stage for six genes chosen from the 21-somite microarray results: *aldh1a2*, *rcan3*, *bmp3*, *hsp90a.1*, *unc45b*, and *fgfrl1a *(Table 1, Figure 2). Such results identify a specific expression domain within the paraxial mesoderm for *bmp3*, whose whole-embryo expression has not previously been reported (Figure 2e-f). In addition, we confirm paraxial mesoderm expression in *aldh1a2 *(Figure 2a-b) [41], *rcan3 *(Figure 2c-d) [52], *hsp90a*.1 (Figure 2g-h) [42,53], *unc45b *(Figure 2i-j) [54], and *fgfrl1a *(Figure 2k-l) [52]. For all six candidate genes, *in situ *hybridization results corroborate the microarray results in that the tail mesoderm expression in *spt *is lower than its counterpart in the wild type. To our knowledge, this study is the first to report that five of these genes are downstream of *tbx16*; the sixth (*aldh1a2*) was recently shown to be regulated by *tbx16 *and *ntl *based on genomic analyses of T-box binding site sequences [19]. In summary, we have identified six genes acting downstream of *tbx16 *in the posterior mesoderm at the 21-somite stage, only one of which (*aldh1a2*) is embedded within a more globally disrupted signaling pathway (retinoic acid signaling).

As a final exploration of our microarray data, we compared our results with those of another microarray analysis of downstream targets of *tbx16 *that used RNA extracted from whole embryos at 75% epiboly and the Compugen/Sigma-Genosys oligo library [19]. We examined ten genes/probes that were present on both the Compugen and Affymetrix arrays and were shown to be downregulated in *spt *by the Garnett et al (2009) [19] study: *her1*, *pcdh10b*, *egln3*, *wu:fb53f04*, *efnb2b*, *tbx6*, *mespa*, *pcdh8*, *myf5*, and *msgn1*. Although our studies examined different developmental stages and tissues, we expect some consistency between these two datasets. In our 21-somite results, eight of these ten genes are downregulated in *spt*, consistent with Garnett et al. [19]: *her1*, *tbx6*, *mespa*, *myf5*, and *pcdh8 *(all at p ≤ 0.05), *msgn1 *(p = 0.07), *pcdh10b *(p = 0.12) and *efnb2b *(p = 0.7). The remaining two genes -- *egln3 *and *wu:fb53f04 *-- were upregulated (p = 0.09) and not differentially expressed (p = 0.13), respectively. However, we note that *efnb2b*, *egln3*, and *wu:fb53f04 *were not present on our filtered gene list, indicating that they are expressed at extremely low levels at the 21-somite stage in the specific tissues we examined. Thus, the differences between our results and those of Garnett et al. may reflect differences in wild-type expression levels between the different developmental stages examined (75% epiboly and the 21-somite stage). Published expression data are consistent with this; *egln3 *is not expressed at the 21-somite stage [52]. To our knowledge, no relevant expression data exist for *wu:fb53f04 *or *efnb2b*. In our 4/5-somite results, six of the ten genes were not present on the filtered gene list, again suggesting that such genes are not expressed in the intermediate mesoderm at this developmental stage; to our knowledge, no definitive evidence of intermediate mesoderm expression exists for these six genes. Of the remaining four genes, two are downregulated in *spt*, consistent with Garnett et al. (2009): *tbx6 *(p = 0.4) and *msgn1 *(p = 0.3). The remaining two are upregulated: *pcdh10b *(p = 0.59) and *wu:fb53f04 *(p = 0.11). This discrepancy may reflect (1) noise in our 4/5-somite microarray data, or (2) biological differences in the effects of *spt *on these targets at different developmental stages and/or in different tissues. In summary, our study and that of Garnett et al. partially corroborate one another, although more detailed analysis of wild-type expression of the Garnett et al. candidates in the tissues we examined would be required to fully integrate our results. More generally, studies looking at downstream targets of *tbx16 *throughout development using a variety of techniques will be required to gain a comprehensive understanding of *tbx16*'s role in mesoderm development.

## Discussion

### *tbx16/spt *and Wnt Signaling

To our knowledge, our results are the first to specifically demonstrate that Wnt signaling lies downstream of *tbx16*. More generally, such results are broadly consistent with several recent studies examining Wnt signaling, T-box genes, and blood development. Analyses identifying direct targets of both *tbx16 *and its close T-box family members *ntl *and *bra *[19,20] have confirmed that *tbx16 *and Wnt co-regulate gene expression that drives mesoderm development [55]. Using microarray analyses of RNA from *tbx16 *and *ntl *morpholino-injected embryos at 75% epiboly, in combination with binding site prediction and genome searches, Garnett et al. (2009) [19] identified another T-box gene, *tbx6*, as a direct target of both *tbx16 *and *ntl*. Notably, the *tbx16 *and *ntl *binding sites were interspersed with Tcf/Lef-binding sites that are crucial for *tbx6 *expression to be responsive to Wnt signaling [56]. Thus, *tbx6 *is directly regulated by both *tbx16 *and Wnt signaling, as well as *ntl*; other putative *tbx16*/Wnt co-regulated targets were also identified, although their detailed investigation has not yet been carried out [19]. Martin and Kimelman (2008) revealed positive regulatory feedback between *ntl/bra *and canonical Wnt signaling in zebrafish mesoderm progenitors, demonstrating another interaction between canonical Wnt signaling and T-box genes. We have identified a novel way in which *tbx16 *and Wnt signaling interact, contributing to the emerging picture of T-box/Wnt interactions that regulate gene expression in the developing mesoderm.

Wnt signaling plays several known roles during the development of red blood cells, acting on different cell populations at stages spanning mesoderm induction to primitive erythroid specification. Such roles have been documented in zebrafish and other organismal and embryonic stem cell (ES) experimental systems [49,50,57]. Lengerke et al. (2008) showed that, in murine ES cells and zebrafish, Wnt signaling, in conjunction with BMP, activates the Cdx-Hox pathway and that this BMP -- Wnt -- Cdx -- Hox sequence is required for hemangioblast specification [48]. Similarly, Nostro et al. (2008) demonstrated that canonical Wnt signaling, BMP signaling, and Activin/Nodal signaling work in concert to specify Flk1+ mesoderm (which includes hemangioblast cells) in murine ES [49]. Most relevant to our results, they also found that Wnt signaling, in the context of VEGF stimulation, plays an indispensable role in the specification of primitive erythroid progenitors from Flk1+ cells; in contrast, neither BMP nor Activin/Nodal signaling was required at this stage [50]. Cheng et al. (2008) also demonstrated that canonical Wnt signaling is required for the establishment of the primitive erythroid lineage from the hemangioblast and that Notch signaling inhibits primitive erythroid development by inhibiting the Wnt pathway [47]. Taken together, these studies underscore the important point that Wnt signaling acts multiple times on different cell populations during development of primitive red blood cells.

Identifying and integrating all roles for Wnt signaling during hematopoiesis into a larger regulatory network that includes additional pathways (e.g. *trf3 *-- *mespa *-- *cdx4 *[58]) and individual hematopoietic genes (e.g. *gfi1.1*, *survivin *[59,60]) remains an important goal [61,62]. Embryonic stem cell studies allow the identification of Wnt pathway effects at discrete time points in development; however, our microarray analysis targeting a specific tissue (the intermediate mesoderm) at a specific developmental stage (4/5-somites) allows even finer-scale analysis because we can map Wnt pathway activity to specific tissue types *in vivo*. This allows us to integrate additional types of developmental data with microarray and ES results to generate more refined hypotheses that address both temporal and spatial aspects of *tbx16 */Wnt function in the developing embryo. For example, cell transplantation studies in zebrafish embryos have demonstrated that (1) the embryonic microenvironment for red blood cell formation in the intermediate mesoderm requires an interaction between intermediate mesoderm and the adjacent paraxial mesoderm, and (2) *tbx16 *is required cell-autonomously in the intermediate mesoderm in order to properly respond to the paraxial mesoderm signal [22]. Existing expression data in zebrafish suggest the hypothesis that the BMP -- Wnt -- Cdx -- Hox pathway identified by Lengerke et al. (2008) [48] may correspond to some aspect of paraxial mesoderm signal production (or competence enabling signal production) in zebrafish. Specifically, *cdx4 *and *cdx1a*, the caudal type homeobox transcription factors involved in primitive hematopoiesis in zebrafish, are both primarily expressed in the paraxial mesoderm [63,64], as are the posterior *hox *genes under the transcriptional regulation of the *cdx *genes [64]. In contrast, the *tbx16-*dependent Wnt signaling identified by our GSEA analysis is likely part of the response to this as-yet-unknown signal in the intermediate mesoderm and may correspond to the Wnt-dependent transition from Flk1+ cells to erythroid progenitors identified in murine ES cells [48,50]. Additionally, Garnett et al. (2009) [19] identified *cdx4 *as a putative target of *tbx16 */Wnt co-regulation, which suggests the hypothesis that *tbx16 *may be part of the BMP -- Wnt -- Cdx -- Hox pathway [49] that may function in zebrafish paraxial mesoderm. Because *spt *embryos lack trunk paraxial mesoderm, transplantation studies have been unable to ascertain whether *tbx16 *functions in the trunk paraxial mesoderm beyond controlling the correct migration of precursor cells during gastrulation to include any more direct role(s) in signal production [22]. Other types of experimental manipulations will be required to rigorously test the hypothesized roles for *tbx16 *and Wnt signaling in both paraxial mesoderm signal production and intermediate mesoderm signal reception generated by incorporating our microarray results with other developmental genetic, genomic, and ES studies.

In summary, previous research has suggested (1) a role for Wnt signaling in specification of both the hemangioblast and the primitive erythroid progenitor lineage, as well as (2) combined regulatory interactions between *tbx16 *and Wnt during diverse stages of mesoderm development. Our results suggesting that *tbx16 *regulates Wnt signaling during the specification of red blood cell progenitors in the zebrafish intermediate mesoderm are thus consistent with previous studies, but add a new level of detail. Such detail was made possible through gene expression analyses targeted to a specific tissue and developmental stage, informed by cell transplantation studies in live embryos. Thus, embryological manipulation and genomics can be integrated to generate novel insights in developmental genomics.

Our GSEA looked for effects of *spt *on a set of genes that included both canonical and non-canonical Wnt pathway members; thus, we tested the effects of *tbx16 *on both canonical and non-canonical Wnt signaling simultaneously. The results demonstrate that both canonical and non-canonical pathways are disrupted. More specifically, the leading edge subset of genes, whose expression levels drive the signal of Wnt upregulation in *spt*, contains Wnt ligands and Frizzled receptors implicated in both signaling pathways (e.g. canonical *wnt8 *and *fzd10*, non-canonical *wnt11 *and *fzd7*). These results, too, are consistent with recent ES studies implicating both canonical and non-canonical Wnt signaling during hematopoiesis. Vijayaragavan et al. (2009) revealed a role for non-canonical Wnt signaling in human ES during Stage I of hematopoiesis, which encompasses hemangioblast formation, as well as canonical Wnt signaling during Stage II, which includes formation of committed hematopoietic progenitors; other studies have also demonstrated early roles for non-canonical Wnt signaling during blood formation [65,66]. More generally, work continues to demonstrate that many pathway members act in both canonical and non-canonical signaling [67-69], suggesting that the traditionally separate canonical and non-canonical Wnt signaling pathways are better represented as an integrated Wnt signaling network [62,70]. Thus, our finding that both canonical and non-canonical components are impacted in *spt *intermediate mesoderm is not surprising and may lend support to an integrated Wnt signaling network acting in zebrafish hematopoiesis.

Although our GSEA results revealed an upregulation of Wnt pathway/network components, predicting the effects of such upregulation on Wnt target genes is not straightforward. Within any signaling network, individual components have very different effects on downstream target gene expression; both activators and inhibitors exist, and components are integrated through both positive and negative feedback loops [71-73] as well as more complicated, dynamic interactions [62]. Thus, our results should be interpreted as a demonstration that Wnt signaling is disrupted; more targeted analyses of expression and function of network components and downstream targets are required to elucidate the specific effects of Wnt signaling disruption on hematopoietic gene expression.

Wnt signaling is not significantly disrupted in the 21-somite tail tissue analysis. This finding is consistent with previous studies demonstrating that canonical Wnt signaling (specifically *wnt3a *and *wnt8*) is required for tail development in zebrafish [74]; given the relatively normal tail somites and presomitic mesoderm present in the *spt *mutant, relatively unperturbed Wnt signaling is not surprising. Furthermore, knocking out *wnt8 *expression in *spt *embryos results in the loss of all tail somites; this suggests that Wnt signaling necessary for posterior somite formation remains intact in *spt *mutants, although Wnt signaling, *tbx16*, and *ntl *interact to generate both trunk and tail mesoderm [55]. Finally, segmentation clock gene expression is intact in *spt *[16]; given the role of Wnt signaling in regulating the segmentation clock [75], intact Wnt signaling in posterior *spt *somites is not unexpected.

### *tbx16/spt *and Retinoic Acid Signaling

Our GSEA results are also broadly consistent with several studies linking *tbx16 *with retinoic acid signaling and intermediate mesoderm differentiation. Garnett et al. (2009), as did we, identified *aldh1a2*, an enzyme that catalyzes the synthesis of retinoic acid, as a likely direct target of *tbx16 *based on the large number of T-box binding sites upstream, and within the intronic sequences, of the gene [19]. Reduced expression of *aldh1a2 *in somitic and intermediate mesoderm has also been reported in both *spt *and *spt/ntl *mutant embryos [76]. Notably, recent studies have identified a prominent role for retinoic acid signaling, controlled by expression of *aldh1a2 *(synthesis) and *cyp26a1 *(degradation), in patterning the zebrafish pronephros, also derived from the intermediate mesoderm [77]. Such retinoic acid signaling is localized along the anterior/posterior axis by *cdx *genes [77]. The downstream targets of retinoic acid in the intermediate mesoderm, and how they contribute to specification of pronephric or hematopoietic fate, are just beginning to be understood [78,79]; BMP signaling has also been shown to contribute to hemato-vascular vs. pronephric fate commitment [80]. Our result that both retinoic acid and Wnt signaling are disrupted in *spt *intermediate mesoderm suggests the additional hypotheses that (1) retinoic acid regulates Wnt signaling, or that (2) Wnt regulates retinoic acid signaling; the latter has been shown previously in zebrafish during gastrulation [81] and patterning of the neuroectoderm [82]. Our result that retinoic acid signaling continues to be disrupted at the 21-somite stage in the developing tail somites is consistent with previous studies showing that *aldh1a2 *expression contributes to both somite size [83] and symmetry [84], as the anterior-most somites formed in *spt *are somewhat irregular [14]. More generally, our results suggest that the effects of *tbx16 *on retinoic acid signaling may persist both spatially and temporally throughout mesoderm differentiation in the developing embryo.

The retinoic acid leading edge subsets of genes, whose expression levels drive the signal of retinoic acid signaling downregulation in both the intermediate mesoderm (4/5-somite stage) and tail (21-somite stage), contain both retinoic acid synthesis (e.g. *aldh1a2*, *rdh1l*) and degradation (e.g. *cyp26a1*) enzymes. Again, the specific effects of such perturbations on downstream targets of retinoic acid signaling are difficult to predict. Our results should be interpreted to reveal a general disruption of retinoic acid signaling in *spt*, the details of which will require further analyses of pathway and target gene expression and function.

## Conclusions

Our study examined the effects of the *spt *mutation on zebrafish embryonic gene expression profiles at two developmental stages and discrete embryonic locations (4/5-somite intermediate mesoderm and 21-somite anterior tail tissue) using microarrays. We performed both GSEA aimed at identifying disrupted signaling pathways as well as analyses to identify individual, differentially expressed genes between *spt *and wild-type tissue. Our results demonstrate that retinoic acid signaling is under the control of *tbx16 *at both developmental stages/tissues. In addition, we reveal that Wnt signaling is under the control of *tbx16 *in the zebrafish intermediate mesoderm at the 4/5-somite stage. This suggests a novel role for Wnt signaling in zebrafish primitive red blood cell formation, adding to an emerging picture of the many roles of Wnt signaling during hematopoiesis. We also identify novel *tbx16 *targets outside of both pathways. Our results contribute to a growing body of research aimed at identifying T-box target genes as well as elucidating the genetic regulation of embryonic red blood cell formation in vertebrates.

## Authors' contributions

All authors contributed to the design of the study. RLM performed the microarray experiments and analyses. CH performed the *in situ *hybridization experiments and analyses. All authors contributed to the writing of the manuscript and approved its final form. The authors declare no competing financial or other interests in relation to this work.

## Supplementary Material

Additional file 1 **Gene sets used for GSEA**. A spreadsheet listing all of the genes in all of the gene sets used for GSEA. Click here for file

Additional file 2 **Leading edge subsets**. A spreadsheet listing the leading edge subsets of genes resulting from GSEA for significantly up- or downregulated gene sets. ** indicates genes that were eliminated when genes present in multiple gene sets were excluded from analysis. Click here for file

Additional file 3 **Significantly differentially expressed genes between *spt *and wild-type**. A spreadsheet listing the significantly differentially expressed genes between spt and wild-type. Click here for file
